# Use of the lambda Red recombinase system to rapidly generate mutants in *Pseudomonas aeruginosa*

**DOI:** 10.1186/1471-2199-9-20

**Published:** 2008-02-04

**Authors:** Biliana Lesic, Laurence G Rahme

**Affiliations:** 1 Department of Surgery, Harvard Medical School and Massachusetts General Hospital, Boston, MA 02114, USA; 2 Shriners' Research Institute, Boston, MA 02114, USA; 3 Department of Microbiology and Molecular Genetics, Harvard Medical School, Boston, MA 02115, USA

## Abstract

**Background:**

The Red recombinase system of bacteriophage lambda has been used to inactivate chromosomal genes in various bacteria and fungi. The procedure consists of electroporating a polymerase chain reaction (PCR) fragment that was obtained with a 1- or 3-step PCR protocol and that carries an antibiotic cassette flanked by a region homologous to the target locus into a strain that expresses the lambda Red recombination system.

**Results:**

This system has been modified for use in *Pseudomonas aeruginosa*. Chromosomal DNA deletions of single genes were generated using 3-step PCR products containing flanking regions 400–600 nucleotides (nt) in length that are homologous to the target sequence. A 1-step PCR product with a homologous extension flanking region of only 100 nt was in some cases sufficient to obtain the desired mutant. We further showed that the *P. aeruginosa *strain PA14 non-redundant transposon library can be used in conjunction with the lambda Red technique to rapidly generate large chromosomal deletions or transfer mutated genes into various PA14 isogenic mutants to create multi-locus knockout mutants.

**Conclusion:**

The lambda Red-based technique can be used efficiently to generate mutants in *P. aeruginosa*. The main advantage of this procedure is its rapidity as mutants can be easily obtained in less than a week if the 3-step PCR procedure is used, or in less than three days if the mutation needs to be transferred from one strain to another.

## Background

The availability of an increasing number of sequenced genomes has generated the need for the development of efficient methods that will enable functional analysis of newly identified genes. The construction of knockout mutants by gene replacement has traditionally been a time consuming process because it requires several subcloning steps. A more time efficient mutagenesis method that does not require cloning was developed recently and has been used in various bacteria and fungi [1-3]. The methodology was first described in *E. coli *[2] and *Aspergillus nidulans *[1]; and subsequently applied successfully to *Yersinia *[4,5], *Salmonella *[6,7], *Shigella *[7] and *Serratia *[8].

The procedure involves the deletion of chromosomal genes via homologous recombination between the chromosomal region of interest and a polymerase chain reaction (PCR)-product that contains an antibiotic cassette flanked by a region of homology with the target DNA. In *E. coli*, a linear DNA is obtained in a 1-step PCR using primers that contain a region that is homologous with a 36-nucleotide (nt) region of the target gene. An efficient recombination between the PCR product and the chromosome is achieved by induction of the lambda phage Red operon. The Red operon encodes the nuclease inhibitor Redγ(*gam*) and the site specific recombinases Redα(*exo) *and Redβ(*bet)*, which mediate homologous recombination [9]. Although molecular biology techniques used in *E. coli *are often applicable to other bacteria, adaptations are frequently required. For example in *Yersinia pseudotuberculosis*, 55-nt homology extensions were generally not sufficient to allow recombination, while *Y. pseudotuberculosis *PCR products with extensions of approximately 500-nt, generated using a 3-step PCR procedure, could trigger reproducibly gene disruption [4].

Here we describe an adaptation of the lambda Red-based methodology for use with *P. aeruginosa*, in which chromosomal single gene deletions were generated using a 3-step PCR product containing 600- to 400-nt flanking regions that are homologous to the target sequence. We further examined the feasibility of using 1-step PCR product with only 100-nt homology extension for obtaining mutants. Finally we tested the ability of this method to delete large chromosomal regions.

## Results

### Gene disruption using a 3-step PCR product

**Figure 1:**
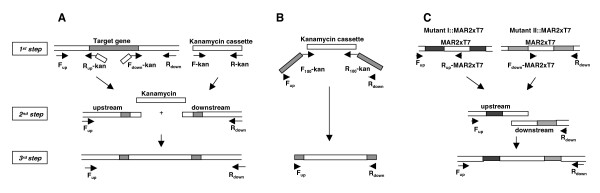
**Principles of 3-step and 1-step PCR**. **A) Single gene deletion using a 3-step PCR**. The first step involves amplifying independently the upstream and downstream regions of the target gene and the kanamycin resistance cassette. Then the three fragments obtained are mixed together and a third amplification performed to augment the yield of the product. **B) Single gene deletion using a 1-step PCR**. The first step is performed using pUC4K (Genbank X06404) as template and a mixture of long and short primers. The long primers *(*F_100_-kan/R_100_-kan) contain at their 5' extremity 100-nt homology to the flanking regions of the target gene and at their 3' extremity 22–24 nt homology to the kanamycin resistance cassette. The short primers F_up_/R_down _are homologous to the 5' extremities of the long primers. **C) Deletion of large chromosomal region using a 3-step PCR**. The first step requires the amplification of MAR2xT7 transposon with the upstream and downstream region of genes of interest. Two transposon mutants are used as template to generate the up and down regions. Then the two fragments are mixed together to generate the desired PCR product and a third step performed to augment the yield of the product.

The mutagenesis was carried out by electroporating a polymerase chain reaction (PCR)-product that contains an antibiotic cassette flanked by sequences homologous to the targeted DNA into a strain expressing the lambda Red operon. The presence of the specific PCR product promotes the deletion of the chromosomally located targeted region via homologous recombination between the genomic region and the flanking sequences of the PCR product. In order to use this system in *P. aeruginosa*, we first cloned the lambda Red operon into the pUCP18 vector. We also cloned the *sacB *gene into this vector to enable a rapid cure of the plasmid following mutagenesis. The generated vector is called pUCP18-RedS (Genbank EU073163) (Additional file 1). Initially, the *pqsC *gene was selected for knockout because the mutant phenotype can be differentiated from the wild-type strain by its inability to produce the *Pseudomonas aeruginosa *blue pigmented phenazine, pyocyanin, lack of which results in a yellowish culture (unpublished data). We used a 3-step PCR procedure to generate a fragment containing the kanamycin resistance cassette flanked by regions surrounding the *pqsC *gene (Fig 1A). In the first step, *P. aeruginosa *genomic DNA was used to amplify the upstream and downstream regions of the target sequence with primers F_up_pqsC/R_up_pqsC-kan and F_down_pqsC-kan/R_down_pqsC (all primer sequences are shown in Additional file 2). In each pair, one primer contains at its 5' end a 20 bp-region homologous to the kanamycin cassette. Simultaneously, the pUC4K plasmid was used to amplify the kanamycin resistance gene. In the second step, the three fragments and primers F_up_pqsC/R_down_pqsC were mixed and a product containing the upstream, kan and downstream regions was obtained. The third step is performed with primers F_up_pqsC/R_down_pqsC to obtain a large quantity of PCR product.

**Figure 2:**
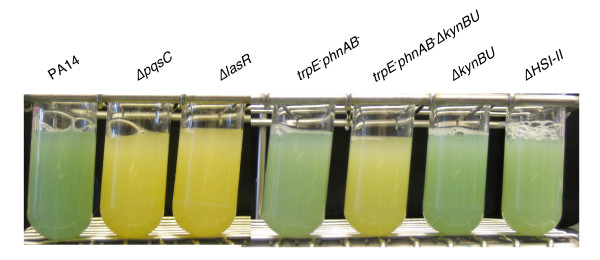
**Δ*pqsC*, Δ*lasR *and *trpE*^-^*phnAB*^-^Δ*kynBU *mutants do not produce pyocyanin**. The production of pyocyanin, the *P. aeruginosa *blue phenazine pigment, is assessed in the mutants generated in this study after overnight growth in LB at 37°C.

**Figure 3:**
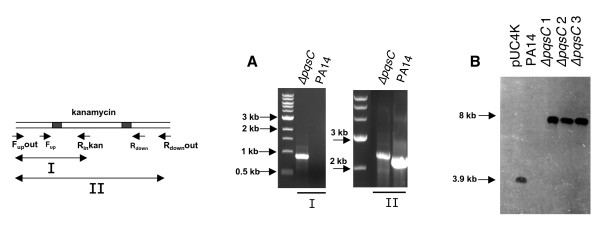
**Verification of the Δ*pqsC *mutant by PCR and Southern**. **A) Verification by PCR**. To confirm that the *pqsC *gene is mutated, two primer pairs F_up_out-pqsC/R_in_-kan and F_up_out-pqsC/R_down_out-pqsC were used, with F_up_out-pqsC and R_down_out-pqsC hybridizing outside the DNA region used for the recombination event. Primers F_up_out/R_in_-kan allow DNA amplification only if the kanamycin cassette is inserted, while the primer pair F_up_out/R_down_out amplify a fragment of different size when mutant or wild type genomic DNA is used. **B) Verification by Southern**. The *Hind*III-digested total genomic DNA of three Δ*pqsC *generated mutants was hybridized using the kanamycin cassette as a probe. One band of 8016 bp is obtained when the cassette is inserted into *pqsC*. The *Hind*III-digested pUC4K and PA14 genomic DNA was used as positive and negative controls, respectively.

We first assessed whether flanking regions of ~600-nt were sufficient to enable homologous recombination between the target gene and the PCR product, as this is generally sufficient when a typical suicide vector is used. Primers F_up_pqsC/R_up_pqsC-kan and F_down_pqsC-kan/R_down_pqsC were designed to amplify regions 651-nt upstream and 610-nt downstream of *pqsC*, and to leave intact the 5' and 3' ends of the gene in the resulting recombinant mutant (Fig. 1A). The 2.2 kb PCR fragment obtained was electroporated into PA14/pUCP18-RedS previously induced by arabinose for the expression of the lambda Red proteins. Aliquots of 2 or 4 μg of electroporated DNA allowed the selection of 10–20 Km^R ^recombinants; that colony yield was approximately doubled when 8 μg of DNA was used. All Km^R ^colonies obtained were analyzed by assessing their ability to produce pyocyanin. Approximately 80% of the colonies potentially carried the insertion, as revealed by their inability to produce pyocyanin (Fig. 2). The correct integration of the kanamycin cassette was then confirmed by PCR in five independent colonies. Figure 3A shows results obtained for one representative Δ*pqsC *recombinant. A PCR product of 868 bp was obtained using primers F_up_out-pqsC/R_in_kan (Additional file 2) and the mutant genomic DNA as template, while no PCR product was obtained when the wild type genomic DNA was used. In addition when the wild type and Δ*pqsC *genomic DNA were used as template, primers F_up_out-pqsC/R_down_out-pqsC (Additional file 2) amplified a product of 2757 bp and 2550 bp (720 bp of *pqsC *was replaced by 927 bp of the kanamycin cassette) respectively (Fig 3A). The correct and unique insertion of the kanamycin resistance cassette was verified by Southern. *Hind*III-digested genomic DNA of three independent colonies was hybridized with the 5' end of the kanamycin cassette, and only one band of 8016 bp was obtained confirming that the cassette was inserted into *pqsC *(Fig 3B). Finally, to ensure inclusion of proper borders in the inserted kanamycin cassette, 700-bp regions upstream and downstream of the cassette were sequenced. The sequences indicated proper insertion of the cassette and that no mutations were incorporated during the 3-step PCR procedure as the flanking sequences were identical to the original.

**Figure 4:**
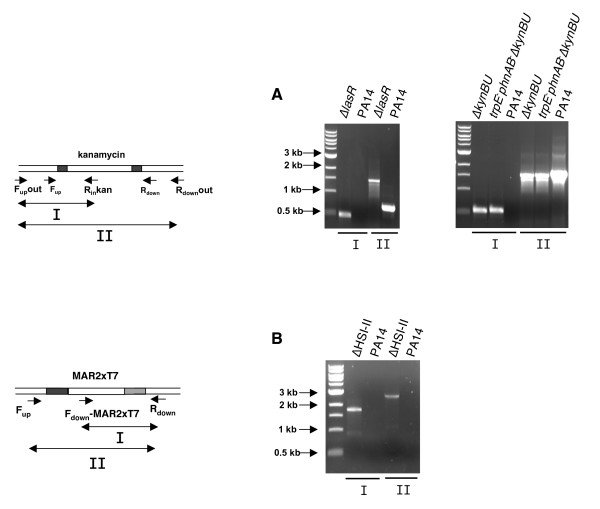
**Verification of the Δ*lasR*, Δ*kynBU*, *trpE*^-^*phnAB*^-^Δ*kynBU *and ΔHSI-II mutants by PCR**. **A. Verification of single gene deletion**. To confirm that the target gene is mutated, two primer pairs F_up_out/R_in_-kan and F_up_out/R_down_out were used. The Δ*lasR *mutant was verified with primer pairs F_up_out-lasR/R_in_-kan and F_up_out-lasR/R_down_out-lasR, Δ*kynBU *and *trpE*^-^*phnAB*^-^Δ*kynBU *with F_up_out-kynBU/R_in_-kan and F_up_out-kynBU/R_down_out-kynBU. **B) Verification of deletion of large chromosomal region**. To confirm that the MAR2xT7 transposon replaced the 24 kb HSI-II locus, primer pairs F_down_-MAR2xT7/R_down_-PA14_42880 and F_up_-PA14_43100/R_down _PA14_42880 were used. No amplification is achieved when wild type genomic DNA is used.

Using 3-step PCR, we also successfully obtained two supplementary mutants: Δ*lasR *and Δ*kynBU*. The Δ*lasR *mutant was generated using 490-nt upstream and 445-nt downstream flanking homologous regions resulting in the replacement of 93 bp of *lasR *by the kanamycin cassette. As expected the *lasR *mutant generated was defected in pyocyanin production (Fig 2). Five independent colonies were analyzed and the correct insertion was confirmed in all five colonies using primers F_up_out-lasR/R_in_kan and F_up_out-lasR/R_down_out-lasR (Additional file 2 and Fig. 4A). The Δ*kynBU *mutant was generated in both wild type and *trpE*^-^*phnAB*^- ^backgrounds using 471-nt upstream and 419-nt downstream flanking homologous regions. In these mutants 1041 bp region of *kynBU *was replaced by the 927 bp in length kanamycin cassette. The *trpE*^-^*phnAB*^-^Δ*kynBU *mutants were functionally validated also using pyocyanin as readout. In fact, *P. aeruginosa *is unable to produce pyocyanin if the bacterium is impaired in its capacity to synthesize 4-hydroxyl-quinolines (HAQs). Recently it was reported that two distinct pathways supply anthranilate as the precursor of HAQs [10]. Anthranilate can be either derived from the conversion of chorismate to anthranilate by an anthranilate synthase: TrpEG and PhnAB; or produced by the degradation of tryptophan through the kynurenine pathway: KynBU. While *kynBU *and *trpE*^-^*phnAB*^- ^mutants were still able to produce pyocyanin in rich medium (Fig 2) due to the fact that the anthranilate or kynurenine pathways supplied anthranilate respectively, no trace of pyocyanin was present in any *trpEphnAB*^-^Δ*kynBU *recombinants tested (Fig 2). Also, DNA amplification was performed in five independent Δ*kynBU *and *trpEphnAB*^-^Δ*kynBU *colonies using primer pairs F_up_out-kynBU/R_in_kan and F_up_out-kynBU/R_down_out-kynBU (Additional file 2) and confirmed the insertion of the kanamycin cassette in all five Δ*kynBU *and *trpEphnAB*^-^Δ*kynBU *recombinants (Fig 4A). Note that PCR products of similar size were obtained with primers F_up_out-kynBU/R_down_out-kynBU when wild type or mutant genomic DNA was used as templates, as 1024 bp of *kynBU *were replaced by 927 bp of kanamycin cassette. Altogether these results demonstrate that the lambda Red technique is an efficient system for generating mutants in *P. aeruginosa*.

### Gene disruption using a 1-step PCR product

The mutagenesis process could be simplified by using a PCR fragment with a shorter region of homology to the target gene. This would enable the product to be obtained in a 1-step PCR using long primers that are homologous to the kanamycin resistance cassette at their 3' ends and homologous to the target gene at their 5' ends. Thus in order to reduce the length of homologous extension necessary to select for *P. aeruginosa *recombinants, the following four sets of primers were designed to amplify the *pqsC*::kan locus: F-pqsC_600_/R-pqsC_600_, F-pqsC_300_/R-pqsC_300_, F-pqsC_150_/R-pqsC_150 _and F-pqsC_100_/R-pqsC_100 _(Additional file 2). The PCR products generated contained the antibiotic cassette flanked with 600, 300, 150 or 100-nt homologous regions to the *pqsC*-surrounding region; when 8 μg of each product was electroporated, 35, 50, 60 and 40 Km^R ^colonies were obtained, respectively, and out of those 30, 46, 50 and 34 positive recombinants were identified based on their inability to produce pyocyanin, which corresponds to a 83–92% positive rate. Five recombinants from each transformation event were analyzed by PCR and the expected products were obtained systematically using primers F_up_out-pqsC/R_in_kan and F_up_out-pqsC/R_down_out-pqsC (Additional file 2). These results show that in the case of *pqsC *mutagenesis the number of positive recombinants was not dependent on the length of the homologous region used and that a 100-nt homologous sequence was sufficient to allow recombination.

To further test the feasibility of 1-step PCR for this application, we designed and purchased primers (Sigma Aldrich) that contain at their 5' extremity 100-nt homology to the flanking regions of *kynBU (*F-kynBU_100_-kan/R-kynBU_100_-kan) or *lasR *(F-lasR_100_-kan/R-lasR_100_-kan) and at their 3' extremity 22–24 nt homology to the kanamycin resistance cassette (Additional file 2). The kanamycin resistance cassette was amplified using pUC4K as the template and a mixture of two sets of primers: F_100_-kan/R_100_-kan and F_up_/R_down _(Fig 1B). The resultant PCR product was used for electroporation. Δ*kynBU *recombinants, but not Δ*lasR *mutants, were obtained using 8 μg of PCR product and confirmed by PCR as described above. Our inability to obtain the Δ*lasR *mutant with this protocol indicates that the 1-step PCR method cannot be considered highly reliable and that 100 nt is likely close to the minimal length of homology needed.

### Transfer of deletion to other mutant backgrounds

The lambda Red methodology was combined with 1-step PCR to transfer a knockout mutation from the original strain in which it was generated to other strains. We amplified the *lasR*::kan locus using the DNA of the previously obtained mutant as the template and the primer pair F-lasR/R-lasR. The amplification generated a PCR product containing 490-nt and 445-nt upstream and downstream flanking target regions. Eight-microgram aliquots of PCR product were electroporated into three PA14 isogenic mutants (Δ*mvfR*, Δ*pqsE *and Δ*pqsA*), and all three strains successfully acquired the mutated locus, which was demonstrated by DNA amplification using primer pairs F_up_out-lasR/R_in_kan and F_up_out-lasR/R_down_out-lasR.

A comprehensive non-redundant PA14 transposition mutant library was recently constructed by random insertion of the MAR2xT7 transposon which confers gentamycin-resistance [11]. We tested whether the lambda Red methodology would allow the transfer of loci where the MAR2xT7 transposon has been randomly inserted. The gentamycin cassette that had been inserted into *pqsC *was amplified using primers F-pqsC_600_/R-pqsC_600 _to generate a product with 379-nt upstream and 471-nt downstream *pqsC*-surrounding regions. Similar to the deletion experiments above, 2, 4 or 8 μg samples of DNA were electroporated into PA14/pUCP18-RedS and yielded 14, 14 and 50 Gm^R ^recombinants, respectively. Five recombinants were tested for the inability to produce pyocyanin in cultures and the presence of the MAR2xT7 transposon was checked by PCR amplification using primers F-pqsC_600_/R-pqsC_600_, respectively (data not shown). These results demonstrate that the lambda Red technique can be used in conjunction with a mutant library to achieve rapid transfer of mutated genes into various PA14 isogenic mutants and thereby enable the creation of multi-locus knockout mutants.

### Deletion of a large DNA region using the lambda Red system

Lambda Red-based methodology has the potential of enabling large regions of chromosomal DNA, including large gene clusters and operons and pathogenicity islands, to be deleted. We examined whether the lambda Red system could be also used to delete the entire *P. aeruginosa *HSI-II locus [12], an approximately 24 kb genomic region encoding a putative type VI secretion system located between PA14_43110 and PA14_42880 genes. The PCR procedure followed to obtain the PCR product necessary to delete the HSI-II locus is presented in Fig 1C. First, genomic DNA of mutants of the PA14 non-redundant transposon library: PA14_43100::MAR2xT7 and PA14_42880::MAR2xT7 were used as template to generate the PCR fragment containing the gentamycin cassette flanked by the HSI-II locus-borders. Primers F_up_-PA14_43100/R_up_-MAR2xT7 were designed to amplify MAR2xT7 and the 890 nt upstream sequence when inserted into PA14_43100; while F_down_-MAR2xT7/R_down_-PA14_42880 were used to amplify MAR2xT7 and the 955 nt downstream sequence when inserted into PA14_42880 (Additional file 2). In a second PCR-step, the resulting upstream and downstream fragments were mixed with the primers F_up_-PA14_43100 and R_down_-PA14_42880 and a PCR product containing MAR2xT7 flanked by the borders of the HSI-II locus obtained. PCR product aliquots of 2, 4 or 8 μg were electroporated into PA14/pUCP18-RedS, and 256, 280 and 300 recombinants were selected on gentamycin, respectively. All mutants tested were producing pyocyanin (Fig 2). DNA amplification using primers F_up_-PA14_43100/R_up_-MAR2xT7 and F_up_-PA14_43100/R_down_-PA14_42880 confirmed deletion of the target region in 5 independent colonies; Fig 4B shows the PCR products obtained for one representative recombinant. While no DNA amplification with the primers F_up_-PA14_43100/R_down_-PA14_42880 flanking the HSI-II locus was obtained when wild type genomic DNA was used as a template, a fragment of 2844 bp was amplified when mutant genomic DNA was used (Fig 4B).

## Discussion

The principal advantage of lambda Red-based mutagenesis is its rapidity. The technique can be performed in less than a week if the 3-step PCR procedure is used, and in less than three days if just the transfer of an existing mutated locus is needed. The protocol has been also further simplified by the use of Choi *et al*'s method for preparing electrocompetent cells [13].

Our results demonstrated that 100-nt homology between the PCR product and the target gene is sufficient in some cases to produce recombination. Nevertheless to maximize the chances of obtaining mutants, the 3-step PCR technique should be used to generate a product with 600- to 400-nt homology extension to the target gene. Also, note that fragments of the same length may have different efficiency of recombination rates due to differing sequence contexts.

The technique has yet never failed; however further experiments should be conducted to additionally test the ability of this methodology to transfer genes marked with an antibiotic cassette from PA14 to various non-isogenic *P. aeruginosa *strains.

To simplify the process, transposon mutants in the PA14 transposon mutant library can serve as templates for amplification of genes of interest already interrupted by an inserted MAR2xT7 transposon carrying the gentamycin cassette. We routinely use this method to produce multi-locus knockout mutants. Moreover, PA14 transposon mutant library can also serve for the rapid deletion of large chromosomal regions. The PAO1 tet^R ^transposon mutant library is an additional useful resource [14]. Thus both libraries together with any mutant that carries a selective marker can be used to generate easily and rapidly multiple-mutation strains. To allow the construction of multiple mutations in the same chromosome, this method can be used in conjunction with previously described methods for marker excision (Flp/Frt) [15], which would allow recycling of the same selection marker in the same strain.

## Conclusion

The lambda Red-based technique can be used efficiently and rapidly to generate mutants in *P. aeruginosa*. Chromosomal single gene deletions were generated using PCR product containing flanking regions that are homologous to the target sequence. To maximize the efficiency of mutants' generation, PCR products with 600- to 400-nt homology extension to the target gene should be used. This technique, in conjunction with previously generated *P. aeruginosa *transposon libraries, can also serve to delete large chromosomal regions.

## Methods

### Strains and growth conditions

*Pseudomonas *and *E. coli *strains were grown in Luria Bertani medium (LB) at 37°C with aeration and when necessary *pseudomonas *cultures were supplemented with kanamycin (400 μg/ml), gentamycin (15 μg/ml) or carbenicillin (300 μg/ml). *Pseudomonas *strains used in this study include Δ*pqsA *[16], Δ*pqsE *[17], Δ*mvfR *[18], PA14_42880::MAR2xT7 and PA14_43100::MAR2xT7 [11]. The *trpE-phnAB- *double mutant was constructed in this study: 1293 bp of *phnA *and 300 bp of *phnB *were deleted by allelic exchange in the auxotroph *trpE*::Mar2xT7 mutant background. The vector pKOBEG-*sacB *[4] contains the Red operon expressed under the control of the arabinose inducible pBAD promoter and the *sacB *gene that is necessary to cure the plasmid [2,4]. Since pKOBEG-*sacB *was not capable of replication in *P. aeruginosa*, the Red operon-*araC *fragment obtained after digestion with *Kpn*I and *Hind*III was cloned into the multi clonal site of the *E. coli *– *P. aeruginosa *shuttle vector pUCP18 (Genbank U07164) [19] to create the plasmid pUCP18-Red. In addition, the *Nde*I *sacB *fragment from pKOBEG-*sacB *was cloned into pUCP18-Red, previously digested with *Nde*I, to create pUCP18-RedS (Genbank EU073163) (Additional file 1). *P. aeruginosa *recombinants were easily cured from pUCP18-RedS by streaking the mutant strains on NaCl-free LB agar plates supplemented with 10% sucrose.

### Southern Blot

Approximately 5 μg of DNA from *P. aeruginosa *and 1 μg of pUC4K DNA was digested by *Hind*III. The 5' end of the kanamycin cassette was amplified using primers 5'-gccacgttgtgtctcaaaat-3' and 5'-gcatttctttccagacttgttc-3', and the PCR product was labeled using the ECL kit (Amersham) and used for hybridization following manufacturer's instructions.

### PCR

All PCRs were performed using High Fidelity Taq polymerase (Roche), 1 μM of each primer, 100 μM of dNTPs. DMSO 8% was added to the reaction when *P. aeruginosa *genomic DNA was used as template. Most of the primers were generated by the Massachusetts General Hospital DNA oligo core facility. The long primers were obtained from Sigma Aldrich.

A kanamycin resistance cassette with long flanking regions homologous to the target gene was generated by a previously described 3-step PCR protocol [4,20] with minor modifications. *P. aeruginosa *genomic DNA was first used as a template to amplify the regions flanking the target gene with the primers F_up_/R_up_-kan and F_down_-kan/R_down _(Additional file 2 and Fig. 1A). R_up_-kan and F_down_-kan contain, respectively, at their 5' extremity 19 and 22-nt regions that are homologous to the 5' and the 3' terminals of the kanamycin resistance gene. The plasmid pUC4K [21] was used as a template to amplify the kanamycin cassette with the primers F-kan/R-kan (Additional file 2). In a 2^nd ^PCR, 10 ng, 25 ng or 50 ng of each of the three fragments were mixed with the primers F_up_/R_down_. A third PCR was finally performed using again the F_up_/R_down _primers and the product of the second PCR as the template in order to increase the yield of the sought band (Fig. 1A). Five hundred microliters (10 reactions) of the 3^rd ^PCR were ethanol precipitated, dissolved in 10 μl of water and dialyzed for 1 h to eliminate salt that can interfere with electroporation.

The 1-step *kynBU *or *lasR *PCR products were generated using pUC4K (Genbank X06404) as template with a mixture of long and short primers (Fig 1B). The long primers contained at their 5' extremity 100-nt homology to the flanking regions of *kynBU (*F-kynBU_100_-kan/R-kunBU_100_-kan) or *lasR *(F-lasR_100_-kan/R-lasR_100_-kan) and at their 3' extremity 22–24 nt homology to the kanamycin resistance cassette. The short primers F-kynBU/R-kunBU and F-lasR/R-lasR were homologous to the 5' extremities of the *kynBU *or the *lasR *long primers, respectively.

The DNA concentration was measured by spectrophotometer at 260 nm and the DNA molecular weight was visually confirmed on agarose gel.

### Gene disruption

*P. aeruginosa *PA14/pUCP18-RedS was grown in LB with carbenicillin to an optical density at 600 nm (OD_600_) of 0.4 at 37°C. Then cells were induced for 1.5 h by treatment with 0.2% L-arabinose; the bacteria were rendered electrocompetent by four washings with 10% sucrose at room temperature and were condensed to a concentration of about 10^11 ^/ml. Electroporation was carried out using 100 μl of bacteria solution and no more than 10 μl of DNA. The electroporated cells were diluted in 2 ml LB and incubated for 2 h at 37°C. Transformants were selected by plating the electroporated cells on antibiotic-imbued plates and further verified by PCR for the correct insertion using two set of primers F_up_out/R_in_kan and F_up_out/R_down_out, with the latter pair hybridizing outside the DNA region used for the recombination event.

## Abbreviations

Polymerase Chain Reaction (PCR), nucleotide (nt), base pair (bp), kan (kanamycin), Kanamycin resistant (Km^R^).

## Authors' contributions

BL performed all experiments. BL and LGR designed experiments and wrote the paper. BL and LGR have read and approved the final version of the manuscript.

## Supplementary Material

Additional File 1 Schematic map of pUCP18-RedS. The schematic representation of pUC18-RedS (Genbank EU073163) includes the Red operon (*gam*, *bet*, *exo*) – *araC *fragment, *sacB *encoding the levansucrase, and *bla *encoding the resistance to ampicillin. Click here for file

Additional File 2 Primers used in this study. The table lists all primers used in the various mutagenesis steps described in this paper. Click here for file
