# Supplementary figures and images for: Use of the lambda Red recombinase system to rapidly generate mutants in Pseudomonas aeruginosa
Source: BMC Mol Biol. 2008 Feb 4;9:20. doi: 10.1186/1471-2199-9-20 (PMC2287187; doi:10.1186/1471-2199-9-20)

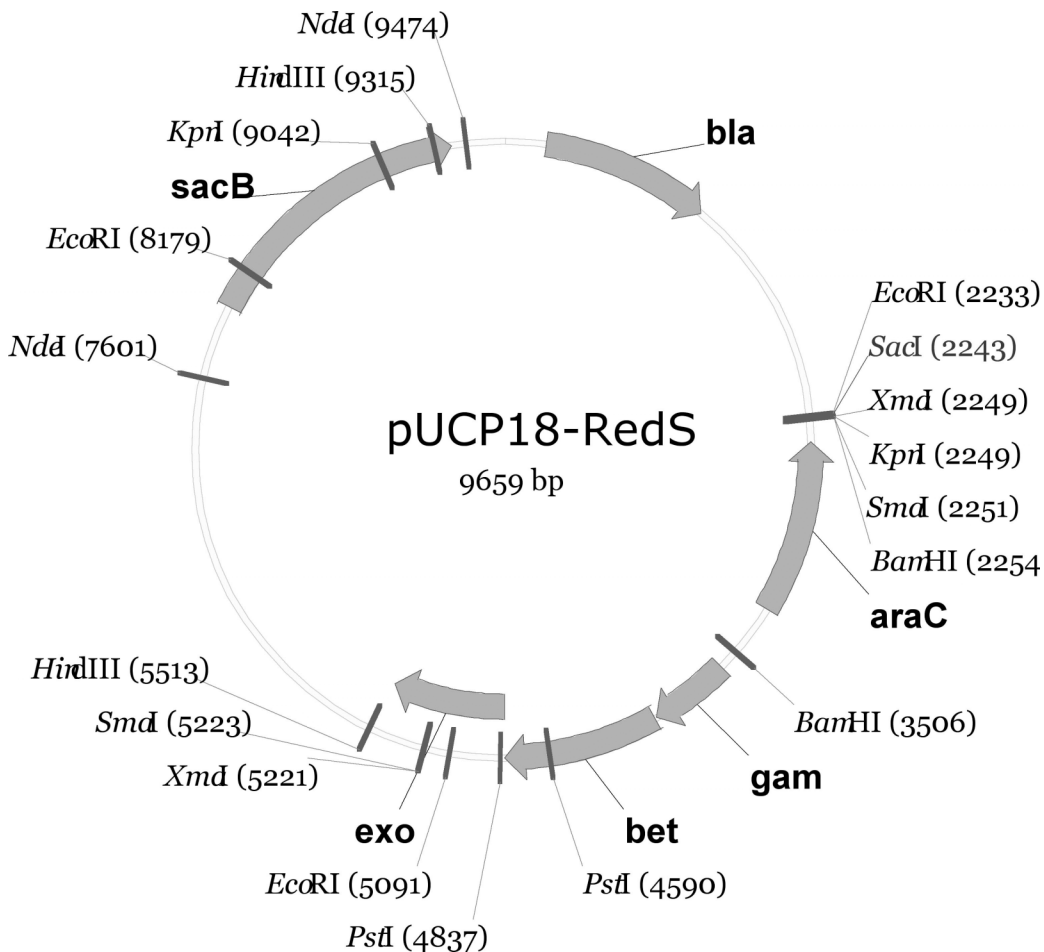

Supplement: Additional File 1 — Schematic map of pUCP18-RedS. The schematic representation of pUC18-RedS (Genbank EU073163) includes the Red operon (gam, bet, exo) – araC fragment, sacB encoding the levansucrase, and bla encoding the resistance to ampicillin. [file 1471-2199-9-20-S1.pdf]
