# Supplementary material for: Use of the lambda Red recombinase system to rapidly generate mutants in Pseudomonas aeruginosa
Source: BMC Mol Biol. 2008 Feb 4;9:20. doi: 10.1186/1471-2199-9-20 (PMC2287187; doi:10.1186/1471-2199-9-20)
Supplement: Additional File 2 — Primers used in this study. The table lists all primers used in the various mutagenesis steps described in this paper. [file 1471-2199-9-20-S2.doc]

**Additional file 2**: Primers used in this study

| **Primers** | **Sequence** |
| --- | --- |
| F-kan  R-kan | 5’-aaagccacgttgtgtctcaaaatc-3’  5’-gcgctgaggtctgcctcgtgaaga-3’ |
| F_up_pqsC  R_up_pqsC-kan | 5’-gcgaatccagccagggattggaagtgga-3’  3’-gattttgagacacaacgtgaactcgttctcgccgttc-3’ |
| F_down_pqsC-kan  R_down_pqsC | 5’-gctcgatgagtttttctaatcagataccggcgtgatgatctcc-3’  5’-gaggatctgcccgcgagccatctgcaag-3’ |
| F_up_outpqsC  R_down_outpqsC | 5’-agcgaccagggctatcgcaacgt-3’  5’-agcagtccgtcttcgagactct-3’ |
| R_in_kan | 5’-tcctatggaactgcctcggt-3’ |
| F_up_lasR  R_up_lasR-kan | 5’-gccaactctatagagtgggctgactggac-3’  5’-gattttgagacacaacgtggactcgtgctgctttcgcgtctggtagat-3’ |
| F_down_lasR-kan  R_down_lasR | 5’-gctcgatgagtttttctaatcatggaagcggaaaaccgggccga-3’  5’-aatggcgagaacctgcccttccctatat-3’ |
| F_up_out-lasR  R_down_out-lasR | 5’-tgtgcctttccggcacaac-3’  5’-atggcttcacacgagagaacacagc-3’ |
| F_up_kynBU  R_up_kynBU-kan | 5’-atgacttcgctccgctactgg-3’  5’-gattttgagacacaacgtgtcgagggtcttggagtgct-3’ |
| F_down_kynBU-kan  R_down_kynBU | 5’-gctcgatgagtttttctaatcaagcgtgtctggcaaccgctg-3’  5’-gtgaagccgaagcgcaggat-3’ |
| F_up_out-kynBU  R_down_out-kynBU | 5’-attcgctggatttccggagaaaaac-3’  5’-gaagcgggtgtacagcggggt-3’ |
| F-pqsC_600_  R-pqsC_600_ | 5’-ttccgtcgcggtgcgctggg-3’  5’-gctggatcaggcaggcctggga-3’ |
| F-pqsC_300_  R-pqsC_300_R | 5’-ttcgaactggcgtcgcaactg-3’  5’-tcgacgtgatagcgggtgcgt-3’ |
| F-pqsC_150_  R-pqsC_150_ | 5’-atgcataaggtcaaactggca-3’  5’-cacaccagcacctcgcccaatt-3’ |
| F-pqsC_100_  R-pqsC_100_ | 5’-atccggtgttcgctgcggtac-3’  5’-ccggccatgacgatacgatc-3’ |
| F-kynBU_100_-kan  R-kunBU_100_-kan | 5’-attcgctggatttccggagaaaaacaatgacttcgctccgctactgggacatcagtcccgccc tcgacccgagcacgccgacctggcccggcgacacgccaaagccacgttgtgtctcaaaatc-3’  5’-aacgatcaccttgcgccctggcgcttcttcctcctggatccgcagggcggcgctgagcacc ttgaacaggttgatcgaggtggtgtcggtgatcaccacttctgattagaaaaactcatcg-3’ |
| F-kynBU  R-kunBU | 5’-attcgctggatttccggagaaaaac-3’  5’-aaacgatcaccttgcgccctgg-3’ |
| F-lasR_100_-kan  R-lasR_100_-kan | 5’-cttggttgacggttttcttgagctggaacgctcaagtggaaaattggagtggagcgccatcctg cagaagatggcgagcgaccttggattctcgaagatcaaagccacgttgtgtctcaaaatc-3’  5’-gaggtcacaccgaacttccgccgaatatttcccatatggaagttcacattggcttccgagcagtt gcagataaccgatatctcccaactggtcttgccgattctgattagaaaaactcatcg-3’ |
| F-lasR  R-lasR | 5’-cttggttgacggttttcttgagctgga-3’  5’-gaggtcacaccgaacttccgccgaata-3’ |
| F_up_-PA14_43100  R_up_-Mar2xT7 | 5’-gcgcgcccagcgcttcctgcaat-3’  5’- caaagttaggtggctcaagtatg-3’ |
| F_down_-Mar2xT7  R_down_-PA14_42880 | 5’- ggtcagcagctcctcgccggtga -3’  5’- ttaggtggcggtacttgggtcg -3’ |
